# Molecular Genetic Markers in Acute Myeloid Leukemia

**DOI:** 10.3390/jcm4030460

**Published:** 2015-03-12

**Authors:** Sophia Yohe

**Affiliations:** Department of Laboratory Medicine and Pathology, Divisions of Hematopathology and Molecular Genetic Pathology, University of Minnesota, MMC Box 609 Mayo, 420 Delaware St. SE. Minneapolis, MN 55455, USA; E-Mail: yohe0001@umn.edu; Tel.: +1-612-273-3098; Fax: +1-612-624-6662

**Keywords:** acute myeloid leukemia (AML), gene mutation, *FLT3*-ITD, *NPM1*, *CEBPA*

## Abstract

Genetics play an increasingly important role in the risk stratification and management of acute myeloid leukemia (AML) patients. Traditionally, AML classification and risk stratification relied on cytogenetic studies; however, molecular detection of gene mutations is playing an increasingly important role in classification, risk stratification, and management of AML. Molecular testing does not take the place of cytogenetic testing results, but plays a complementary role to help refine prognosis, especially within specific AML subgroups. With the exception of acute promyelocytic leukemia, AML therapy is not targeted but the intensity of therapy is driven by the prognostic subgroup. Many prognostic scoring systems classify patients into favorable, poor, or intermediate prognostic subgroups based on clinical and genetic features. Current standard of care combines cytogenetic results with targeted testing for mutations in *FLT3*, *NPM1*, *CEBPA*, and *KIT* to determine the prognostic subgroup. Other gene mutations have also been demonstrated to predict prognosis and may play a role in future risk stratification, although some of these have not been confirmed in multiple studies or established as standard of care. This paper will review the contribution of cytogenetic results to prognosis in AML and then will focus on molecular mutations that have a prognostic or possible therapeutic impact.

## 1. Introduction

There is a well-established role for genetic classification of acute myeloid leukemia (AML) into different prognostic groups. Traditionally, this classification has relied on detection of large chromosomal abnormalities by cytogenetics; however, detection of smaller scale mutations is playing an increasingly important role in classification and prognostication of AML. These mutations do not take the place of cytogenetic testing results but play a complementary role to help refine prognosis, especially within specific AML subgroups.

With the exception of acute promyelocytic leukemia, therapy for AML is not targeted and the intensity of therapy is driven by the prognostic subgroup. Many prognostic scoring systems classify patients into favorable, poor, or intermediate prognosis based on clinical and cytogenetic features. Research on molecular testing has generally tried to refine the prognosis of intermediate cases or to find mutations that explain why some patients in a favorable prognosis category have resistant disease. If identified, these patient could potential receive more aggressive therapy upfront. This paper will briefly review the contribution of cytogenetic results to prognosis in AML and then will focus on molecular mutations that change prognostic subgrouping. Gene mutations that appear to have prognostic effect but have not been confirmed in multiple studies or established as standard of care will also be explored.

## 2. Genetics and AML Classification

The current World Health Organization (WHO) 2008 classifies AML based on patient history, morphologic findings, and the presence or absence of specific genetic abnormalities. Genetic abnormalities play the biggest role in two categories: AML with recurrent genetic abnormalities and AML with myelodysplasia related changes (AML-MRC) [1]. (Table 1) AML-MRC can also be diagnosed in patients with a history of a myelodysplastic syndrome (MDS) or based on the presence of significant morphologic dysplasia in two cell lineages at the time of AML diagnosis. However, current treatment guidelines for AML use only a subset of the AML-MRC genetic abnormalities to guide therapy in the absence of a history of MDS (Table 2) [2]. The presence of morphologic dysplasia alone does not affect therapy.

## 3. Cytogenetic Abnormalities and Prognosis

Most of the prognostic cytogenetic abnormalities in AML are either chromosomal rearrangements or large genomic deletions. (Table 2) Acute promyelocytic leukemia with t(15;17) and the core binding factor leukemias with inv(16)/t(16;16) or t(8;21) have a better prognosis. In contrast, complex or monosomal karyotypes, deletions of chromosomes 5 or 7, and some other specific chromosomal rearrangements have a poorer prognosis. Other changes including normal cytogenetics, t(9;11) and isolated +8 have an intermediate prognosis. However, the presence of certain molecular mutations may modify these prognostic groups. Isolated *NPM1* or biallelic *CEBPA* mutations improve the prognosis of AML with normal cytogenetics from intermediate to favorable; whereas a *FLT3* ITD changes it to poor. The presence of a *KIT* mutation in core binding factor leukemia worsens the prognostic category to intermediate.

**Table 1:** Genetic abnormalities that affect acute myeloid leukemia (AML) classification.

AML with Recurrent Genetic Abnormalities	AML with Myelodysplasia Related Changes
*RUNX1*-*RUNX1*T1 t(8;21)(q22;q22)	Complex karyotype (≥3 unrelated abnormalities)
CBFB-MYH11 inv(16)(p12.1q22) or t(16;16)(p13.1;q22)	−7/del(7q), −5/del(5q)
PML-RARA t(15;17)(q22;q12)	−13/del(13q), del(11q), del(12p)/t(12p), del(9q)
*MLL*T3-*MLL*/*KMT2A* t(9;11)(q22;q23)	i(17q)/t(17p), idic(X)(q13)
DEK-NUP214 t(6;9)(p23;q34)	t(5;12)(q33;p12), t(5;7)(q33;q11.2) t(5;17)(q33;p13), t(5;10)(q33;q21)
RPN-EVI1 inv(3)(q21q26.2) or t(3;3)(q21;q26.2)	t(1;3)(p36.3;q21.2), t(3;5)(q25;q34)
RBM15-MKL1 t(1;22)(p13;q13)	t(11;16)(q23;p13.3) *, t(3;21)(q26.2;q22.1) *
*NPM1* gene mutation (provisional entity)	t(2;11)(p21;q23) *
Mutated *CEBPA* (provisional entity)	

* Rule out therapy related AML before using any of these three translocations to make a diagnosis of AML with myelodysplasia related changes.

**Table 2:** Cytogenetic and molecular findings used in risk stratification for AML.

Risk	Cytogenetics	Molecular
Favorable	inv(16) or t(16;16)	Normal cytogenetics with:
	t(8;21)	Isolated biallelic *CEBPA* mutation
	t(15;17)	*NPM1* mutation without *FLT3* ITD
Intermediate	Normal cytogenetics	*KIT* mutation in core binding factor leukemia: inv(16) or t(16;16) t(8;21)
	Isolated +8	
	t(9;11)	
	Other non-good and non-poor changes	
Poor	Complex (≥3 clonal abnormalities)	Normal cytogenetics with: *FLT3* ITD
	Monosomal karyotype *	
	−5/−5q or −7/−7q	
	11q23 rearrangements other than t(9;11)	
	inv(3) or t(3;3)	
	t(6;9)	
	t(9;22)	

* Monosomal: ≥2 monosomies or 1 monosomy and additional 1 or more structural abnormalities (Breems JCO 2008; 26:4791); ITD: internal tandem duplication. (Adapted with permission from the NCCN Clinical Practice Guidelines in Oncology (NCCN Guidelines^®^) for Acute Myeloid Leukemia V.1.2015 © National Comprehensive Cancer Network, Inc 2014. All rights reserved. Accessed January 13, 2015. To view the most recent and complete version of the guideline, go online to NCCN.org. NATIONAL COMPREHENSIVE CANCER NETWORK^®^, NCCN^®^, NCCN GUIDELINES^®^, and all other NCCN Content are trademarks owned by the National Comprehensive Cancer Network, Inc.)

## 4. Established Gene Mutations Associated with Prognosis

Refining the prognosis for AML in the cytogenetic intermediate risk category has received the most attention. As this group is heterogeneous, the best treatment for an individual patient in the intermediate risk category is uncertain. Favorable risk patients are treated with standard chemotherapy while patients in the poor risk category should undergo allogeneic hematopoietic stem cell transplant. However, 40%–50% of adult AML falls into the intermediate category and most of these have a normal karyotype. *FLT3*, *NPM1* and *CEBPA* mutations were the first to be found useful in helping stratify cytogenetically intermediate risk patients with a normal karyotype. Mutations in *KIT* help to refine prognosis in core binding factor leukemia. 

### 4.1. FLT3 (Fms-like Tyrosine Kinase 3)

*FLT3* is a receptor tyrosine kinase involved in hematopoiesis and commonly mutated in AML. There are two common mutations that occur in *FLT3*: an internal tandem duplication (ITD) in the juxtamembrane domain and a point mutation of the tyrosine kinase domain (TKD). Both mutations lead to constitutive activation; however only the *FLT3* ITD is definitively associated with a poorer prognosis. About 20% of all AMLs harbor a *FLT3* ITD mutation, but the mutation is more common in AML with t(15;17) and AML with a normal karyotype (cytogenetically normal AML or CN-AML), accounting for approximately 30% of these cases [3,4]. AML with a normal karyotype and *FLT3* ITD mutation has a poorer prognosis [3,4,5]. 

Testing of patients by PCR followed by size analysis, reveals variability in the size of the *FLT3* ITD, the number of *FLT3* ITD mutations, and the amount of *FLT3* ITD mutation compared to wild type. Some of these have prognostic implications. Studies have shown that patients with a higher *FLT3* ITD mutant:wild type allelic ratio have a worse prognosis than patients with a lower ratio [6,7]. Although the ratio may reflect disease burden to a certain extent, a high allelic ratio of >0.5 (or ratio ≥1 using area under the curve) is presumed to be due to biallelic *FLT3* ITD mutations in at least a subset of the blasts [7,8]. Despite the prognostic impact, current risk stratification does not include the allelic ratio. Approximately 14%–25% of *FLT3* ITD positive patients will have more than one *FLT3* ITD mutation, in these cases the mutant:wild type ratio of the most prevalent mutation should be used for the allelic ratio [6,7,9]. Most studies have not shown a prognostic effect of having multiple *FLT3* ITD mutations [6,7,8,10]. The *FLT3* ITD size can vary from a few base pairs to over 1000 base pairs [7]. A correlation between size and prognosis has been demonstrated in some studies but not others [6,7,11,12,13]. 

Sequencing of *FLT3* ITD reveals that there is also variability in the site and sequence of the mutations, in fact some mutations are not true tandem duplications and not all *FLT3* ITD are in the juxtamembrane domain. The term *FLT3* length mutation (*FLT3* LM) has been proposed as a more accurate term [7]. Only about two-thirds of *FLT3* mutations are actual duplications while the remaining third are insertions or complex duplications and insertions [7]. Despite the sequence differences, mutations appear to remain in-frame [7,10]. The insertion site of the *FLT3* mutation is highly variable, one study found 91 unique insertion sites in 689 patients [7]. Approximately 30% of *FLT3* ITD occur outside the juxtamembrane domain and instead occur in the first tyrosine kinase domain (TKD1), usually in the β1 sheet [7,10,14]. At least some of these *FLT3* ITD in the TKD1 domain have been shown to lead to constitutive activation [14]. Kayser *et al*. in 2009 and Schlenk have shown worse prognosis with insertion in the TKD1 domain, but the 2012 study by Schnittger did not [7,8,9]. The Schnittger study did show a trend to worse prognosis with a more 3’ location of the insertion and the TKD1 domain is more 3’ than the juxtamembrane domain [7]. Further studies are needed to evaluate the different *FLT3* mutations and insertion sites to determine whether specific mutations have different prognostic impacts.

Allogeneic transplant is usually recommended for *FLT3* ITD positive AML with a normal karyotype; however, even with transplant there is a high risk of relapse. There is also interest in targeting *FLT3* ITD mutations with *FLT3* inhibitors; unfortunately, to date success in this area has been limited [15]. Possible reasons include coexistence or development of *FLT3* TKD mutations, activation of downstream signaling molecules, up-regulation of *FLT3*, or activation of other pathways [15]. 

The less common *FLT3* TKD mutation is found in about 10% of AML and also leads to constitutive activation of *FLT3* [3,4]. However, despite a seemingly similar mechanism of action the *FLT3* TKD has not clearly been shown to have an effect on prognosis. Some studies suggested an adverse prognostic risk; however, other studies have not confirmed this [3,4]. It is unclear at this time whether this mutation is targetable with *FLT3* inhibitors, although some studies suggest that it is not [15]. 

### 4.2. NPM1 (Nucleophosmin 1)

*NPM1* encodes a phosphoprotein that normally shuttles between the nucleus and cytoplasm and plays a role in ribosome biogenesis, centrosome duplication during mitosis, and cell proliferation and apoptosis through p53 and p19Arf [16]. Mutations in *NPM1* occur in the C-terminus of the gene leading to loss of the nucleolar localization signal and gain of a nuclear export signal ultimately leading to cytoplasmic localization of this protein. The most common mutation is a 4 base pair insertion. *NPM1* mutations are found in about 30% of all AML and 50%–60% of AML with a normal karyotype making it the most common genetic mutation in AML [3]. *NPM1* rarely occurs with the any of the recurrent genetic abnormalities, *BCOR*, or *CEBPA* but frequently co-exist with *FLT3*, *DNMT3A*, and IDH [17,18,19]. 

The presence of an *NPM1* mutation in AML with normal karyotype in the absence of a *FLT3* ITD mutation portends a favorable prognosis similar to the core-binding factor leukemias [5,17]. Some studies have suggested that an *NPM1* mutation with a *FLT3* ITD mutation has a prognosis intermediate compared to either mutation in isolation; while some studies suggest this may only be the case when the *FLT3* ITD mutation load is low [6,20]. There is limited data suggesting that the presence of multi-lineage dysplasia or an adverse karyotype do not affect the favorable prognosis of *NPM1* mutations as long as *FLT3* ITD is absent [21,22]. 

### 4.3. CEBPA (CCAAT Enhancer Binding Protein)

*CEBPA* is a transcription factor involved in neutrophil differentiation. *CEBPA* mutations are found in approximately 10% of AML and are more common in AML with a normal karyotype or with 9q deletions [4]. *CEBPA* mutations in AML may be biallelic, which accounts for approximately two-thirds of cases, or monoallelic, accounting for the remaining cases. In AML with a normal karyotype, isolated biallelic *CEBPA* mutations clearly confer a better prognosis, whereas a monoallelic mutation likely does not confer the same favorable prognosis [23,24,25]. A recent meta-analysis does not show a better prognosis with monoallelic *CEBPA* and in long term follow-up, biallelic *CEBPA* mutations show a longer overall survival (9.6 years) *versus* monoallelic *CEBPA* mutations (1.7 years) [23,24]. Biallelic mutations usually include one C-terminus and one N-terminus mutation and lead to absent expression of normal *CEBPA* [26,27]. The truncating N-terminal mutations result in a shortened *CEBPA* protein with a dominant negative effect [28]. The C-terminal mutations decrease dimerization or DNA binding [25]. 

### 4.4. KIT (v-KIT Hardy-Zuckerman 4 Feline Sa12rcoma Viral Oncogene Homolog)

*KIT* is a receptor tyrosine kinase involved in proliferation, differentiation, and survival. *KIT* mutations affect predominantly exons 8 or 17, lead to a gain of function, and occur in 2%–14% of all cases of AML [18,29,30,31]. The incidence of *KIT* mutations is higher in core-binding factor leukemia, being found in about 7%–46% of cases [32,33,34]. The presence of *KIT* mutations in core binding factor leukemia is generally accepted to be associated with a worse prognosis [35,36]. However, some studies have shown this to be the case only in t(8;21) AML [37,38] and other studies have failed to show a prognostic effect at all [18,39,40]. 

## 5. Other Gene Mutations in AML

With the advent of next generation sequencing, the number of genes found to be mutated in AML has drastically increased. However, the significance of many of these gene mutations is unclear as the genes that are independent predictors of poor outcome differ between studies. (Table 3) Some of these differences may be due to the methods used for mutation detection, but often the statistically significant findings are based on a relatively small subset of patients and therefore may not be reproducible. Additionally, a gene found to be significant in one study may not have been tested in earlier or concurrent studies. Many of these significant genes are also mutated in other myeloid neoplasms; therefore, the presence of one of these mutations is not specific or diagnostic of leukemia. 

**Figure 1:**
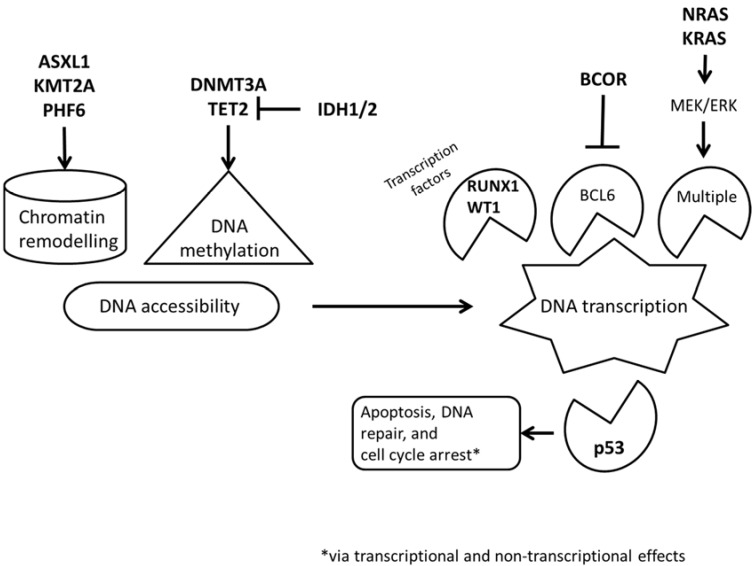
Direct and indirect effects of *ASXL1*, *BCOR*, *DNMT3A*, *IDH1*, *IDH2*, *KMT2A* (*MLL*), *KRAS*, *NRAS*, *PHF6*, *RUNX1*, *TET2*, *TP53*, and *WT1* on DNA transcription.

All of these genes affect transcription either directly or through epigenetic regulation. (See Figure 1) *DNMT3A*, *TET2*, and *IDH1*/2 are involved in DNA methylation. The DNA methyl transferases (DNMT) add a methyl group to CpG islands leading to DNA methylation, the TET proteins convert the methyl group to a hydroxymethyl group. 5-Hydroxymethylation appears to have different effects than methylation and is also an intermediate step to de-methylation. Isocitrate dehydrogenase inhibits TET proteins through 2-hydroxyglutarate. *NRAS*, *KRAS*, *BCOR*, *RUNX1*, and *WT1* all affect transcription. *NRAS* and *KRAS* affect transcription through the MEK/ERK pathway; while *BCOR* affects transcription by repression of BCL6. *RUNX1* and *WT1* are transcription factors; in addition, some *WT1* isoforms appear to regulate mRNA. *ASXL1*, *KMT2A* (*MLL*), and *PHF6* all affect chromatin remodeling. *TP53* is a gatekeeper that monitors DNA repair and regulates apoptosis and the cell cycle. 

The role of testing for these other genes is not well established. Although routine testing of all AML cases is not recommended at this time, testing may be useful to better stratify individual patients. Several studies have proposed alternative stratification of AML patients using some of these genes [19,29,41]. These alternative algorithms risk stratify at least as well as the standard risk stratification given in Table 2 and the scheme proposed by Patel *et al*. appears to perform better than the standard risk stratification [29]. However, these are single studies that need to be confirmed before any of these algorithms are implemented as standard care.

**Table 3:** Other gene mutations in AML.

Gene	Frequency	Effect
*ASXL1*	3%–5%	Associated with MDS, AML-MRC. Worse prognosis [19,29,42,43,44].
*BCOR*	4% CN-AML	Possible worse prognosis [45].
*DNMT3A*	20%	Possible worse prognosis. May respond to high dose anthracyclines [18,29].
*IDH1*	6%–9% adult	Possible worse prognosis [29,46,47,48,49].
	1% pediatric	
*IDH2*	8%–12% adult	Controversial. *IDH2* R140 mutation with *NPM1* associated with a favorable prognosis in one study [29,46,47,48,49].
	1%–2% pediatric	
*MLL*/*KMT2A*	4%–14%	*MLL* PTD shows worse prognosis in CN-AML [18,19,29,30,31].
*NRAS*	8%–13% adult and pediatric	No clear impact on prognosis [50,51].
*KRAS*	2% adult	No clear impact on prognosis [52].
	9% pediatric	
*PHF6*	2%–3%	Associated with adverse outcome [29].
*RUNX1*	5%–18%	Possibly poorer prognosis. May do better with allogeneic transplant [19,29,53].
*TET2*	7%–10% adult	Unclear, some studies show adverse outcome especially in intermediate risk AML with isolated *CEBPA* or *NPM1* [18,29,54,55].
	1.5%–4% pediatric	
*TP53*	2%–9% adult	Unfavorable prognosis [18,19]. Mutations may be germline (Li-Fraumeni syndrome) and this possibility should be considered when testing especially in younger individuals.
	1% pediatric	
*WT1*	4%–11%	Poorer outcome, especially in CN-AML [56,57].

MDS: myelodysplastic syndrome, AML-MRC: acute myeloid leukemia with myelodysplasia related changes, PTD: partial tandem duplication, CN-AML: cytogenetically normal acute myeloid leukemia.

### 5.1. ASXL1 (Additional Sex Combs like Transcriptional Regulator 1)

*ASXL1* encodes a chromatin binding protein, which may enhance or repress gene transcription in localized areas by modification of chromatin structure. *ASXL1* mutations are frequently found in myelodysplastic syndromes (MDS) and in AML but appear to be enriched in secondary AML, AML-MRC, and intermediate risk AML [42,43]. The overall frequency in AML is 3%–5% [18,29,30] but is 11%–17% in intermediate risk AML (including AML with a normal karyotype) [31,58]. *ASXL1* mutations also increase with age, being more prevalent in patients over 60 and quite rare in children (approximately 1%) [58,59,60]. Most studies have shown that *ASXL1* mutations are associated with a worse prognosis; however, studies have not always controlled for a history of MDS or presence of AML-MRC [19,29,44]. *ASXL1* mutation status may change with relapse with both gains and losses of mutations being reported [61]. 

### 5.2. BCOR (BCL6 Corepressor)

The *BCOR* gene is located on the X chromosome and, as its name suggests, plays a role in repression of BCL6 [62]. The BCOR protein interacts with histone deacetylases (HDAC) which may explain its role in AML. *BCOR* mutations in AML have been described in a limited number of studies [45,63,64]. *BCOR* mutations occur in about 4% of CN-AML and frequently coexist with *DNMT3A* mutations [45]. *BCOR* mutations have also been described in 25% of AML cases with trisomy 13 [63]. The effect of *BCOR* mutations in prognosis is unclear at this time. One study showed decreased event free survival but no difference in overall survival in multivariate analysis [45]. 

### 5.3. DNMT3A (DNA Methyltransferase 3A)

*DNMT3A* is a DNA methyltransferase involved in the epigenetic regulation of the genome through methylation. Mutations in *DNMT3A* are quite common in AML, occurring in approximately 20% of patients. The most common mutation is a substitution of the amino acid arginine at position 882 (R882) [65]. *DNMT3A* mutations often co-occur with *FLT3* ITD, *NPM1*, *IDH1*, and *IDH2* mutations but are rare with t(15;17) and core binding factor rearrangements [65]. *DNMT3A* mutations in some studies have been associated with worse prognosis; however, this may be overcome by high dose anthracycline chemotherapy [18,29]. 

### 5.4. IDH1 and IDH2 (Isocitrate Dehydrogenase 1 and 2)

*IDH1* and *IDH2* are genes involved in metabolism that appear to play an epigenetic role in histone and possibly DNA methylation [66]. Mutations in *IDH1* and *IDH2* occur at the active isocitrate binding site and lead to increased level of 2-hydroxyglutarate [67]. IDH mutations often occur with *NPM1* mutations and some studies have shown an impact only with *NPM1* but others have not [29,46,68]. When evaluated together, *IDH1* and *IDH2* mutations have been reported to have a favorable, neutral, and adverse effect on prognosis in AML with a normal karyotype [29,46,47,48]. However, despite an apparently similar biological effect, different mutations may have disparate prognostic impact. This fact makes it difficult to evaluate studies that grouped *IDH1* and *IDH2* mutations and the different *IDH2* mutations together. 

*IDH1* mutations affect either the arginine residue at position 132 or 170 (R132 or R170) and occur in 6%–9% of adult AML cases but only 1% of pediatric AML [29,30,31,46,49,59]. These mutations are exclusive of each other and exclusive of the *IDH2* mutation. When evaluated as a separate group, mutations in *IDH1* appear to have an unfavorable prognosis [49]. 

*IDH2* mutations may affect either the arginine residue at position 140 or 172 (R140 or R172) and occur in 8%–12% of adult AML cases but only 1%–2% of pediatric cases [29,30,31,46,49,59,69]. However, only the R140 mutation appears to have prognostic significance [29,70]. The R140 mutation in *IDH2* has been shown to be associated with a favorable outcome in intermediate risk AML with *NPM1* mutation [29]. 

### 5.5. MLL/KMT2A (Mixed Lineage Leukemia/Lysine (K)-Specific Methyltransferase 2A)

The *MLL* gene (recently renamed to *KMT2A*) is a histone methyltransferase that regulates gene transcription through histone modification. Rearrangements involving *MLL* are well-known to cause acute lymphoblastic leukemia (ALL), AML, or mixed phenotype acute leukemia. However, partial tandem duplications of *MLL* (*MLL* PTD) occur predominantly in AML and are rare in ALL [71]. Approximately 4%–14% of AML cases will have an *MLL* PTD, which has been associated with a poor prognosis especially in AML with a normal karyotype [18,19,29,30,31]. 

### 5.6. NRAS and KRAS (Neuroblastoma RAS Viral (v-ras) Oncogene Homolog and Kirsten Rat Sarcoma Viral Oncogene Homolog)

*KRAS* and *NRAS* belong to the RAS GTPase family of genes. *NRAS* mutations in AML are fairly common being found in 8%–13% of cases in adults and children [17,29,30,31,59]. *KRAS* mutations are less common in adults being found in only 2% of cases but are more common in children where they account for about 9% of cases [29,59]. RAS mutations are more common in core binding factor leukemia, especially inv(16) [38,72]. Although some smaller studies have suggested a worse outcome; in large adult and pediatric studies, *NRAS* mutations have had no clear impact on outcome [50,51]. *KRAS* mutations also do not appear to have an impact on outcome [52].

### 5.7. PHF6 (Plant Homeodomain Finger 6)

*PHF6* is an X-linked gene that appears to play a role in chromatin remodeling although its precise functions have not yet been elucidated [73]. Germline loss of function mutations are associated with X-linked intellectual disability disorders [74]. *PHF6* mutations occur in 2%–3% of adult AML and occur more frequently in males than females [29,75,76]. *PHF6* mutations are associated with adverse outcome in intermediate risk AML patients who are negative for *FLT3* ITD and it has been suggested that mutations in *PHF6* as well as other genes may be useful in stratifying this subgroup of AML patients [29]. Although this study result appears promising, further studies are needed as these conclusions were drawn on a limited number of patients. Only 14 patients had *PHF6* mutations in the test cohort of 398 patients and only 10 patients had *PHF6* mutations and were *FLT3* ITD negative (Patel, *et al*., 2012 supplemental material) [29].

### 5.8. RUNX1 (Runt Related Transcription Factor 1)

*RUNX1* (previously known as AML1) encodes the alpha subunit of core binding factor. Core binding factor is a heterodimer composed of an alpha and beta subunit that is in involved in transcription. Translocations involving *RUNX1* are found in AML with recurrent cytogenetic abnormalities (AML with t(8;21), *RUNX1*-*RUNX1*T1) and also in ALL. Mutations of *RUNX1* also occur in 5%–18% of AML, but are more common in intermediate risk AML and poor risk AML without a complex karyotype [19,29,31,53]. Germline *RUNX1* mutations are found in familial platelet disorder which predisposes to AML and less frequently T-lymphoblastic leukemia (TALL) [77]. Although several studies have shown a poorer prognosis with *RUNX1*, some studies have failed to show an effect [19,29,53]. A study by Gaidzik, *et al*. suggested that patients with *RUNX1* mutations did better with allogeneic transplant compared to autologous transplant or chemotherapy alone [53]. 

### 5.9. TET2 (Tet Methylcytosine Dioxygenase 2)

*TET2* is an epigenetic modifier that converts methylcytosine to 5-hydroxymethylcytosine and plays a role in myelopoiesis. Mutations in *TET2* are found in 7%–10% of adult AML patients and 1.5%–4% of pediatric AML [59,78,79]. Mutations in *TET2* are highly variable and include nonsense mutations, deletions (frameshift and non-frameshift), missense mutations, and splice site mutations. All mutations, however, appear to cause loss of function and decreased hydroxymethylation of DNA [78]. *NPM1* and *TET2* defects are significantly correlated and *FLT3*-ITD and *FLT3*-TKD aberrations are often present together with *TET2* mutations [54,78]. *TET2* and IDH mutations seldom co-existed in the same patient as may be expected since IDH mutations abrogate the activity of *TET2* [31,54]. The frequency of *TET2* mutations in AML increases with age [31]. Of note, *TET2* mutations have been found in elderly females with no clear evidence of hematologic disease [80]. The prognostic effect of *TET2* is unclear with some studies showing an inferior survival in AML with a normal karyotype, especially those with favorable genetic mutations (isolated *CEBPA* and *NPM1*), and other studies showing no effect [18,29,54,55].

### 5.10. TP53 (Tumor Protein p53)

*TP53* is a well-known tumor suppressor gene that regulates the cell cycle in response to cellular stress. Mutations in *TP53* occur in 2%–9% of adult AML and approximately 1% of pediatric AML [18,19,29,59]. *TP53* mutations are highly associated with a complex karyotype and rarely occur with *CEBPA*, *NPM1*, *FLT3* ITD, *ASXL1*, or *RUNX1* mutations [19]. As in other cancers, mutations of *TP53* in AML are associated with an unfavorable prognosis [18,19]. The presence of *TP53* mutation in a young person with AML brings up the possibility of a germline mutation and underlying Li-Fraumeni syndrome. If testing for *TP53* will be performed, the patient should be counselled regarding this possibility. 

### 5.11. WT1 (Wilms Tumor 1)

*WT1* encodes a transcription factor that plays an important role in urogenital development and appears to have a tumor suppressor role in renal tissues but an oncogenic role in leukemia [81]. Overexpression of *WT1* in AML is linked with poor outcome and relapse in several studies especially in AML with a normal karyotype [82,83]. Monitoring levels of *WT1* also has shown usefulness in monitoring for minimal residual disease [84,85]. Mutations in *WT1* also occur, being found 4%–11% of AML cases [29,30,31,43,59]. *WT1* mutations also appear to have an association with poor outcome in AML with a normal karyotype [56,57].

## 6. Conclusions

Genetics play an increasingly important role in the risk stratification and management of AML patients. Current standard of care combines cytogenetic results with testing for mutations in *FLT3*, *NPM1*, *CEBPA*, and *KIT*. The presence of *FLT3* ITD, *NPM1*, or *CEBPA* mutations refines the prognosis of patient with AML with normal karyotype which is normally intermediate risk. *FLT3* ITD modifies the risk to poor, while *NPM1* and biallelic *CEBPA* mutations improve the prognosis to favorable. *KIT* mutations in one of the core binding factor leukemias worsen the prognosis from good to intermediate.

As molecular testing methods advance, routinely testing multiple genes for mutations becomes more feasible and, indeed, gene panels that look for mutations in multiple genes are already available. Mutations in several genes appear to have prognostic impact. However, studies in the literature do not always agree on which mutations have independent prognostic effect and our understanding of the impact of co-existing mutations and the interplay with cytogenetic abnormalities is limited. Mutations in *ASXL1*, *MLL*, *TP53*, and *WT1* have been shown in multiple studies to be independently associated with a poorer prognosis. Mutations in *BCOR*, *DNMT3A*, *IDH1*, *PHF6*, *RUNX1* and *TET2* are possibly associated with a poorer prognosis but have either not been confirmed in multiple studies or have some conflicting results. *KRAS* and *NRAS* mutations do not appear to have an effect on prognosis. As prognosis guides therapy, these gene mutations could play a role in guiding therapy in the future. Two genes appear promising for more specifically guiding therapy in the future. AML with *DNMT3A* mutations may respond better to high dose anthracyclines and AML with *RUNX1* mutations may have better outcomes with allogeneic transplant. These findings are promising that testing for mutations in these additional genes can improve the current risk stratification and patient care; however, they need to be confirmed in additional studies before routine clinical implementation.

## References

[1] Swerdlow S.H., Campo E., Harris N.L., Jaffe E.S., Pileri S.A., Stein H., Thiele J., Vardiman J.W. (2008). Who Classification of Tumours of Hematopoietic and Lymphoid Tissues.

[2] O’Donnel M.R., Tallman M.S., Abboud C.N., Altman J.K., Appelbaum F.R., Arber D.A., Attar E., Borate U., Damon L.E., Gregory K. National comprehensive cancer network: NCCN categories of evidence and consensus. http://www.nccn.org/professionals/physician_gls/categories_of_consensus.asp.

[3] Ofran Y., Rowe J.M. (2013). Genetic profiling in acute myeloid leukaemia—Where are we and what is its role in patient management. Br. J. Haematol..

[4] Martelli M.P., Sportoletti P., Tiacci E., Martelli M.F., Falini B. (2013). Mutational landscape of AML with normal cytogenetics: Biological and clinical implications. Blood Rev..

[5] Whitman S.P., Archer K.J., Feng L., Baldus C., Becknell B., Carlson B.D., Carroll A.J., Mrózek K., Vardiman J.W., George S.L. (2001). Absence of the wild-type allele predicts poor prognosis in adult *de novo* acute myeloid leukemia with normal cytogenetics and the internal tandem duplication of *FLT3*: A cancer and leukemia group B study. Cancer Res..

[6] Gale R.E., Green C., Allen C., Mead A.J., Burnett A.K., Hills R.K., Linch D.C., Party M.R.C.A.L.W. (2008). The impact of *FLT3* internal tandem duplication mutant level, number, size, and interaction with *NPM1* mutations in a large cohort of young adult patients with acute myeloid leukemia. Blood.

[7] Schnittger S., Bacher U., Haferlach C., Alpermann T., Kern W., Haferlach T. (2012). Diversity of the juxtamembrane and TKD1 mutations (exons 13–15) in the *FLT3* gene with regards to mutant load, sequence, length, localization, and correlation with biological data. Genes Chromosomes Cancer.

[8] Schlenk R.F., Kayser S., Bullinger L., Kobbe G., Casper J., Ringhoffer M., Held G., Brossart P., Lübbert M., Salih H.R. (2014). Differential impact of allelic ratio and insertion site in *FLT3*-ITD positive AML with respect to allogeneic transplantation. Blood.

[9] Kayser S., Schlenk R.F., Londono M.C., Breitenbuecher F., Wittke K., Du J., Groner S., Späth D., Krauter J., Ganser A. (2009). Insertion of *FLT3* internal tandem duplication in the tyrosine kinase domain-1 is associated with resistance to chemotherapy and inferior outcome. Blood.

[10] Blau O., Berenstein R., Sindram A., Blau I.W. (2013). Molecular analysis of different *FLT3*-ITD mutations in acute myeloid leukemia. Leuk. Lymphoma.

[11] Meshinchi S., Stirewalt D.L., Alonzo T.A., Boggon T.J., Gerbing R.B., Rocnik J.L., Lange B.J., Gilliland D.G., Radich J.P. (2008). Structural and numerical variation of *FLT3*-ITD in pediatric AML. Blood.

[12] Stirewalt D.L., Kopecky K.J., Meshinchi S., Engel J.H., Pogosova-Agadjanyan E.L., Linsley J., Slovak M.L., Willman C.L., Radich J.P. (2006). Size of *FLT3* internal tandem duplication has prognostic significance in patients with acute myeloid leukemia. Blood.

[13] Ponziani V., Gianfaldoni G., Mannelli F., Leoni F., Ciolli S., Guglielmelli P., Antonioli E., Longo G., Bosi A., Vannucchi A.M. (2006). The size of duplication does not add to the prognostic significance of *FLT3* internal tandem duplication in acute myeloid leukemia patients. Leukemia.

[14] Breitenbuecher F., Schnittger S., Grundler R., Markova B., Carius B., Brecht A., Duyster J., Haferlach T., Huber C., Fischer T. (2009). Identification of a novel type of itd mutations located in nonjuxtamembrane domains of the *FLT3* tyrosine kinase receptor. Blood.

[15] Alvarado Y., Kantarjian H.M., Luthra R., Ravandi F., Borthakur G., Garcia-Manero G., Konopleva M., Estrov Z., Andreeff M., Cortes J.E. (2014). Treatment with *FLT3* inhibitor in patients with *FLT3*-mutated acute myeloid leukemia is associated with development of secondary *FLT3*-tyrosine kinase domain mutations. Cancer.

[16] Falini B., Albiero E., Bolli N., De Marco M.F., Madeo D., Martelli M., Nicoletti I., Rodeghiero F. (2007). Aberrant cytoplasmic expression of C-terminal-truncated NPM leukaemic mutant is dictated by tryptophans loss and a new NES motif. Leukemia.

[17] Schlenk R.F., Döhner K., Krauter J., Fröhling S., Corbacioglu A., Bullinger L., Habdank M., Späth D., Morgan M., Benner A. (2008). Mutations and treatment outcome in cytogenetically normal acute myeloid leukemia. N. Engl. J. Med..

[18] Kihara R., Nagata Y., Kiyoi H., Kato T., Yamamoto E., Suzuki K., Chen F., Asou N., Ohtake S., Miyawaki S. (2014). Comprehensive analysis of genetic alterations and their prognostic impacts in adult acute myeloid leukemia patients. Leukemia.

[19] Grossmann V., Schnittger S., Kohlmann A., Eder C., Roller A., Dicker F., Schmid C., Wendtner C.M., Staib P., Serve H. (2012). A novel hierarchical prognostic model of AML solely based on molecular mutations. Blood.

[20] Schnittger S., Bacher U., Kern W., Alpermann T., Haferlach C., Haferlach T. (2011). Prognostic impact of *FLT3*-ITD load in *NPM1* mutated acute myeloid leukemia. Leukemia.

[21] Falini B., Macijewski K., Weiss T., Bacher U., Schnittger S., Kern W., Kohlmann A., Klein H.U., Vignetti M., Piciocchi A. (2010). Multilineage dysplasia has no impact on biologic, clinicopathologic, and prognostic features of aml with mutated nucleophosmin (*NPM1*). Blood.

[22] Haferlach C., Mecucci C., Schnittger S., Kohlmann A., Mancini M., Cuneo A., Testoni N., Rege-Cambrin G., Santucci A., Vignetti M. (2009). AML with mutated *NPM1* carrying a normal or aberrant karyotype show overlapping biologic, pathologic, immunophenotypic, and prognostic features. Blood.

[23] Li H.Y., Deng D.H., Huang Y., Ye F.H., Huang L.L., Xiao Q., Zhang B., Ye B.B., Lai Y.R., Mo Z.N. (2014). Favorable prognosis of biallelic *CEBPA* gene mutations in acute myeloid leukemia patients: A meta-analysis. Eur. J. Haematol..

[24] Pastore F., Kling D., Hoster E., Dufour A., Konstandin N.P., Schneider S., Sauerland M.C., Berdel W.E., Buechner T., Woermann B. (2014). Long-term follow-up of cytogenetically normal *CEBPA*-mutated AML. J. Hematol. Oncol..

[25] Wouters B.J., Löwenberg B., Erpelinck-Verschueren C.A., van Putten W.L., Valk P.J., Delwel R. (2009). Double *CEBPA* mutations, but not single *CEBPA* mutations, define a subgroup of acute myeloid leukemia with a distinctive gene expression profile that is uniquely associated with a favorable outcome. Blood.

[26] Mueller B.U., Pabst T. (2006). C/EBPalpha and the pathophysiology of acute myeloid leukemia. Curr. Opin. Hematol..

[27] Nerlov C. (2004). C/EBPalpha mutations in acute myeloid leukaemias. Nat. Rev. Cancer.

[28] Pabst T., Mueller B.U., Zhang P., Radomska H.S., Narravula S., Schnittger S., Behre G., Hiddemann W., Tenen D.G. (2001). Dominant-negative mutations of *CEBPA*, encoding CCAAT/enhancer binding protein-alpha (c/EBPalpha), in acute myeloid leukemia. Nat. Genet..

[29] Patel J.P., Gönen M., Figueroa M.E., Fernandez H., Sun Z., Racevskis J., Van Vlierberghe P., Dolgalev I., Thomas S., Aminova O. (2012). Prognostic relevance of integrated genetic profiling in acute myeloid leukemia. N. Engl. J. Med..

[30] Shen Y., Zhu Y.M., Fan X., Shi J.Y., Wang Q.R., Yan X.J., Gu Z.H., Wang Y.Y., Chen B., Jiang C.L. (2011). Gene mutation patterns and their prognostic impact in a cohort of 1185 patients with acute myeloid leukemia. Blood.

[31] Tian X., Xu Y., Yin J., Tian H., Chen S., Wu D., Sun A. (2014). *TET2* gene mutation is unfavorable prognostic factor in cytogenetically normal acute myeloid leukemia patients with *NPM1*^+^ and *FLT3*-ITD^-^ mutations. Int. J. Hematol..

[32] Care R.S., Valk P.J., Goodeve A.C., Abu-Duhier F.M., Geertsma-Kleinekoort W.M., Wilson G.A., Gari M.A., Peake I.R., Löwenberg B., Reilly J.T. (2003). Incidence and prognosis of c-*KIT* and *FLT3* mutations in core binding factor (CBF) acute myeloid leukaemias. Br. J. Haematol..

[33] Beghini A., Ripamonti C.B., Cairoli R., Cazzaniga G., Colapietro P., Elice F., Nadali G., Grillo G., Haas O.A., Biondi A. (2004). *KIT* activating mutations: Incidence in adult and pediatric acute myeloid leukemia, and identification of an internal tandem duplication. Haematologica.

[34] Mrózek K., Marcucci G., Paschka P., Bloomfield C.D. (2008). Advances in molecular genetics and treatment of core-binding factor acute myeloid leukemia. Curr. Opin. Oncol..

[35] Paschka P., Marcucci G., Ruppert A.S., Mrózek K., Chen H., Kittles R.A., Vukosavljevic T., Perrotti D., Vardiman J.W., Carroll A.J. (2006). Adverse prognostic significance of *KIT* mutations in adult acute myeloid leukemia with inv(16) and t(8;21): A cancer and leukemia group B study. J. Clin. Oncol..

[36] Cairoli R., Beghini A., Grillo G., Nadali G., Elice F., Ripamonti C.B., Colapietro P., Nichelatti M., Pezzetti L., Lunghi M. (2006). Prognostic impact of c-*KIT* mutations in core binding factor leukemias: An Italian retrospective study. Blood.

[37] Park S.H., Chi H.S., Min S.K., Park B.G., Jang S., Park C.J. (2011). Prognostic impact of c-*KIT* mutations in core binding factor acute myeloid leukemia. Leuk. Res..

[38] Boissel N., Leroy H., Brethon B., Philippe N., de Botton S., Auvrignon A., Raffoux E., Leblanc T., Thomas X., Hermine O. (2006). Incidence and prognostic impact of c-*KIT*, *FLT3*, and *RAS* gene mutations in core binding factor acute myeloid leukemia (CBF-AML). Leukemia.

[39] Jourdan E., Boissel N., Chevret S., Delabesse E., Renneville A., Cornillet P., Blanchet O., Cayuela J.M., Recher C., Raffoux E. (2013). Prospective evaluation of gene mutations and minimal residual disease in patients with core binding factor acute myeloid leukemia. Blood.

[40] Cairoli R., Beghini A., Turrini M., Bertani G., Nadali G., Rodeghiero F., Castagnola C., Lazzaroni F., Nichelatti M., Ferrara F. (2013). Old and new prognostic factors in acute myeloid leukemia with deranged core-binding factor beta. Am. J. Hematol..

[41] Döhner H., Estey E.H., Amadori S., Appelbaum F.R., Büchner T., Burnett A.K., Dombret H., Fenaux P., Grimwade D., Larson R.A. (2010). Diagnosis and management of acute myeloid leukemia in adults: Recommendations from an international expert panel, on behalf of the european leukemianet. Blood.

[42] Devillier R., Gelsi-Boyer V., Brecqueville M., Carbuccia N., Murati A., Vey N., Birnbaum D., Mozziconacci M.J. (2012). Acute myeloid leukemia with myelodysplasia-related changes are characterized by a specific molecular pattern with high frequency of *ASXL1* mutations. Am. J. Hematol..

[43] Fernandez-Mercado M., Yip B.H., Pellagatti A., Davies C., Larrayoz M.J., Kondo T., Pérez C., Killick S., McDonald E.J., Odero M.D. (2012). Mutation patterns of 16 genes in primary and secondary acute myeloid leukemia (AML) with normal cytogenetics. PLoS One.

[44] Metzeler K.H., Becker H., Maharry K., Radmacher M.D., Kohlschmidt J., Mrózek K., Nicolet D., Whitman S.P., Wu Y.Z., Schwind S. (2011). *ASXL1* mutations identify a high-risk subgroup of older patients with primary cytogenetically normal aml within the eln favorable genetic category. Blood.

[45] Grossmann V., Tiacci E., Holmes A.B., Kohlmann A., Martelli M.P., Kern W., Spanhol-Rosseto A., Klein H.U., Dugas M., Schindela S. (2011). Whole-exome sequencing identifies somatic mutations of *BCOR* in acute myeloid leukemia with normal karyotype. Blood.

[46] Paschka P., Schlenk R.F., Gaidzik V.I., Habdank M., Krönke J., Bullinger L., Späth D., Kayser S., Zucknick M., Götze K. (2010). *IDH1* and *IDH2* mutations are frequent genetic alterations in acute myeloid leukemia and confer adverse prognosis in cytogenetically normal acute myeloid leukemia with *NPM1* mutation without *FLT3* internal tandem duplication. J. Clin. Oncol..

[47] Marcucci G., Maharry K., Wu Y.Z., Radmacher M.D., Mrózek K., Margeson D., Holland K.B., Whitman S.P., Becker H., Schwind S. (2010). *IDH1* and *IDH2* gene mutations identify novel molecular subsets within de novo cytogenetically normal acute myeloid leukemia: A cancer and leukemia group Bstudy. J. Clin. Oncol..

[48] Thol F., Damm F., Wagner K., Göhring G., Schlegelberger B., Hoelzer D., Lübbert M., Heit W., Kanz L., Schlimok G. (2010). Prognostic impact of *IDH2* mutations in cytogenetically normal acute myeloid leukemia. Blood.

[49] Abbas S., Lugthart S., Kavelaars F.G., Schelen A., Koenders J.E., Zeilemaker A., van Putten W.J., Rijneveld A.W., Löwenberg B., Valk P.J. (2010). Acquired mutations in the genes encoding *IDH1* and *IDH2* both are recurrent aberrations in acute myeloid leukemia: Prevalence and prognostic value. Blood.

[50] Berman J.N., Gerbing R.B., Alonzo T.A., Ho P.A., Miller K., Hurwitz C., Heerema N.A., Hirsch B., Raimondi S.C., Lange B. (2011). Prevalence and clinical implications of *NRAS* mutations in childhood AML: A report from the children's oncology group. Leukemia.

[51] Bacher U., Haferlach T., Schoch C., Kern W., Schnittger S. (2006). Implications of *NRAS* mutations in AML: A study of 2502 patients. Blood.

[52] Bowen D.T., Frew M.E., Hills R., Gale R.E., Wheatley K., Groves M.J., Langabeer S.E., Kottaridis P.D., Moorman A.V., Burnett A.K. (2005). *RAS* mutation in acute myeloid leukemia is associated with distinct cytogenetic subgroups but does not influence outcome in patients younger than 60 years. Blood.

[53] Gaidzik V.I., Bullinger L., Schlenk R.F., Zimmermann A.S., Röck J., Paschka P., Corbacioglu A., Krauter J., Schlegelberger B., Ganser A. (2011). *RUNX1* mutations in acute myeloid leukemia: Results from a comprehensive genetic and clinical analysis from the AML study group. J. Clin. Oncol..

[54] Gaidzik V.I., Paschka P., Späth D., Habdank M., Köhne C.H., Germing U., von Lilienfeld-Toal M., Held G., Horst H.A., Haase D. (2012). *TET2* mutations in acute myeloid leukemia (AML): Results from a comprehensive genetic and clinical analysis of the AML study group. J. Clin. Oncol..

[55] Metzeler K.H., Maharry K., Radmacher M.D., Mrózek K., Margeson D., Becker H., Curfman J., Holland K.B., Schwind S., Whitman S.P. (2011). *TET2* mutations improve the new European leukemianet risk classification of acute myeloid leukemia: A cancer and leukemia group B study. J. Clin. Oncol..

[56] Krauth M.T., Alpermann T., Bacher U., Eder C., Dicker F., Ulke M., Kuznia S., Nadarajah N., Kern W., Haferlach C. (2014). *WT1* mutations are secondary events in AML, show varying frequencies and impact on prognosis between genetic subgroups. Leukemia.

[57] Sano H., Shimada A., Tabuchi K., Taki T., Murata C., Park M.J., Ohki K., Sotomatsu M., Adachi S., Tawa A. (2013). *WT1* mutation in pediatric patients with acute myeloid leukemia: A report from the Japanese childhood AML cooperative study group. Int. J. Hematol..

[58] Schnittger S., Eder C., Jeromin S., Alpermann T., Fasan A., Grossmann V., Kohlmann A., Illig T., Klopp N., Wichmann H.E. (2013). *ASXL1* exon 12 mutations are frequent in AML with intermediate risk karyotype and are independently associated with an adverse outcome. Leukemia.

[59] Liang D.C., Liu H.C., Yang C.P., Jaing T.H., Hung I.J., Yeh T.C., Chen S.H., Hou J.Y., Huang Y.J., Shih Y.S. (2013). Cooperating gene mutations in childhood acute myeloid leukemia with special reference on mutations of *ASXL1*, *TET2*, *IDH1*, *IDH2*, and *DNMT3A*. Blood.

[60] El-Sharkawi D., Ali A., Evans C.M., Hills R.K., Burnett A.K., Linch D.C., Gale R.E. (2014). *ASXL1* mutations are infrequent in young patients with primary acute myeloid leukemia and their detection has a limited role in therapeutic risk stratification. Leuk. Lymphoma.

[61] Chou W.C., Huang H.H., Hou H.A., Chen C.Y., Tang J.L., Yao M., Tsay W., Ko B.S., Wu S.J., Huang S.Y. (2010). Distinct clinical and biological features of *de novo* acute myeloid leukemia with additional sex comb-like 1 (*ASXL1*) mutations. Blood.

[62] Huynh K.D., Fischle W., Verdin E., Bardwell V.J. (2000). *BCOR*, a novel corepressor involved in BCL-6 repression. Genes Dev..

[63] Herold T., Metzeler K.H., Vosberg S., Hartmann L., Röllig C., Stölzel F., Schneider S., Hubmann M., Zellmeier E., Ksienzyk B. (2014). Isolated trisomy 13 defines a homogeneous aml subgroup with high frequency of mutations in spliceosome genes and poor prognosis. Blood.

[64] Thota S., Viny A.D., Makishima H., Spitzer B., Radivoyevitch T., Przychodzen B., Sekeres M.A., Levine R.L., Maciejewski J.P. (2014). Genetic alterations of the cohesin complex genes in myeloid malignancies. Blood.

[65] Ibrahem L., Mahfouz R., Elhelw L., Abdsalam E.M., Soliman R. (2015). Prognostic significance of *DNMT3A* mutations in patients with acute myeloid leukemia. Blood Cells Mol. Dis..

[66] Lu C., Ward P.S., Kapoor G.S., Rohle D., Turcan S., Abdel-Wahab O., Edwards C.R., Khanin R., Figueroa M.E., Melnick A. (2012). *IDH* mutation impairs histone demethylation and results in a block to cell differentiation. Nature.

[67] Ward P.S., Cross J.R., Lu C., Weigert O., Abel-Wahab O., Levine R.L., Weinstock D.M., Sharp K.A., Thompson C.B. (2012). Identification of additional *IDH* mutations associated with oncometabolite r(-)-2-hydroxyglutarate production. Oncogene.

[68] Green C.L., Evans C.M., Hills R.K., Burnett A.K., Linch D.C., Gale R.E. (2010). The prognostic significance of *IDH1* mutations in younger adult patients with acute myeloid leukemia is dependent on *FLT3*/ITD status. Blood.

[69] Ho P.A., Kutny M.A., Alonzo T.A., Gerbing R.B., Joaquin J., Raimondi S.C., Gamis A.S., Meshinchi S. (2011). Leukemic mutations in the methylation-associated genes *DNMT3A* and *IDH2* are rare events in pediatric AML: A report from the children’s oncology group. Pediatr. Blood Cancer.

[70] Green C.L., Evans C.M., Zhao L., Hills R.K., Burnett A.K., Linch D.C., Gale R.E. (2011). The prognostic significance of *IDH2* mutations in AML depends on the location of the mutation. Blood.

[71] Burmeister T., Meyer C., Schwartz S., Hofmann J., Molkentin M., Kowarz E., Schneider B., Raff T., Reinhardt R., Gökbuget N. (2009). The *MLL* recombinome of adult CD10-negative B-cell precursor acute lymphoblastic leukemia: Results from the gmall study group. Blood.

[72] Goemans B.F., Zwaan C.M., Miller M., Zimmermann M., Harlow A., Meshinchi S., Loonen A.H., Hählen K., Reinhardt D., Creutzig U. (2005). Mutations in *KIT* and *RAS* are frequent events in pediatric core-binding factor acute myeloid leukemia. Leukemia.

[73] Todd M.A., Picketts D.J. (2012). *PHF6* interacts with the nucleosome remodeling and deacetylation (NuRD) complex. J. Proteome Res..

[74] Lower K.M., Turner G., Kerr B.A., Mathews K.D., Shaw M.A., Gedeon A.K., Schelley S., Hoyme H.E., White S.M., Delatycki M.B. (2002). Mutations in *PHF6* are associated with Börjeson-Forssman-Lehmann syndrome. Nat. Genet..

[75] Yoo N.J., Kim Y.R., Lee S.H. (2012). Somatic mutation of *PHF6* gene in T-cell acute lymphoblatic leukemia, acute myelogenous leukemia and hepatocellular carcinoma. Acta Oncol..

[76] Van Vlierberghe P., Patel J., Abdel-Wahab O., Lobry C., Hedvat C.V., Balbin M., Nicolas C., Payer A.R., Fernandez H.F., Tallman M.S. (2011). *PHF6* mutations in adult acute myeloid leukemia. Leukemia.

[77] Preudhomme C., Renneville A., Bourdon V., Philippe N., Roche-Lestienne C., Boissel N., Dhedin N., André J.M., Cornillet-Lefebvre P., Baruchel A. (2009). High frequency of *RUNX1* biallelic alteration in acute myeloid leukemia secondary to familial platelet disorder. Blood.

[78] Aslanyan M.G., Kroeze L.I., Langemeijer S.M., Koorenhof-Scheele T.N., Massop M., van Hoogen P., Stevens-Linders E., van de Locht L.T., Tönnissen E., van der Heijden A. (2014). Clinical and biological impact of *TET2* mutations and expression in younger adult AML patients treated within the EORTC/GIMEMA AML-12 clinical trial. Ann. Hematol..

[79] Langemeijer S.M., Jansen J.H., Hooijer J., van Hoogen P., Stevens-Linders E., Massop M., Waanders E., van Reijmersdal S.V., Stevens-Kroef M.J., Zwaan C.M. (2011). *TET2* mutations in childhood leukemia. Leukemia.

[80] Busque L., Patel J.P., Figueroa M.E., Vasanthakumar A., Provost S., Hamilou Z., Mollica L., Li J., Viale A., Heguy A. (2012). Recurrent somatic *TET2* mutations in normal elderly individuals with clonal hematopoiesis. Nat. Genet..

[81] Yang L., Han Y., Suarez Saiz F., Saurez Saiz F., Minden M.D. (2007). A tumor suppressor and oncogene: The *WT1* story. Leukemia.

[82] Lyu X., Xin Y., Mi R., Ding J., Wang X., Hu J., Fan R., Wei X., Song Y., Zhao R.Y. (2014). Overexpression of wilms tumor 1 gene as a negative prognostic indicator in acute myeloid leukemia. PLoS One.

[83] Woehlecke C., Wittig S., Arndt C., Gruhn B. (2014). Prognostic impact of *WT1* expression prior to hematopoietic stem cell transplantation in children with malignant hematological diseases. J. Cancer Res. Clin. Oncol..

[84] Rossi G., Carella A.M., Minervini M.M., Savino L., Fontana A., Pellegrini F., Greco M.M., Merla E., Quarta G., Loseto G. (2013). Minimal residual disease after allogeneic stem cell transplant: A comparison among multiparametric flow cytometry, wilms tumor 1 expression and chimerism status (complete chimerism *versus* low level mixed chimerism) in acute leukemia. Leuk. Lymphoma.

[85] Yoon J.H., Kim H.J., Shin S.H., Yahng S.A., Lee S.E., Cho B.S., Eom K.S., Kim Y.J., Lee S., Min C.K. (2013). Serial measurement of *WT1* expression and decrement ratio until hematopoietic cell transplantation as a marker of residual disease in patients with cytogenetically normal acute myelogenous leukemia. Biol. Blood Marrow Transpl..

